# Iodine intake and its association with sociodemographic and dietary factors in Australian preschool children

**DOI:** 10.1007/s00394-026-04004-9

**Published:** 2026-06-19

**Authors:** Marion E. Rogerson, Carley A. Grimes, Ewa A. Szymlek-Gay

**Affiliations:** Institute for Physical Activity and Nutrition (IPAN), School of Exercise and Nutrition Sciences, Deakin University, Melbourne Burwood Campus, 221 Burwood Highway, Burwood, VIC 3125 Australia

**Keywords:** Iodine intake, Preschool children, Plant-based diets, Australia

## Abstract

**Purpose:**

Imbalanced iodine intakes in early childhood may impair growth and neurodevelopment, yet iodine intake in Australian preschoolers remains poorly characterised. This study aimed to assess the prevalence of inadequate and excessive iodine intake, identify sociodemographic and dietary factors associated with iodine intakes, and determine dietary sources of iodine in Australian children aged 2–5 years.

**Methods:**

Data of children aged 2–5 years (*n* = 762) from the 2011–2013 National Nutrition and Physical Activity Survey were analysed. Dietary intake was assessed using up to two 24-h recalls, with usual intakes estimated via the Multiple Source Method. The proportion of children meeting the Estimated Average Requirement for iodine (EAR; 65 µg/day for 1-8-year-olds) or exceeding the Upper Level of Intake for iodine (UL; 200 µg/day for 1-3-year-olds and 300 µg/day for 4-8-year-olds) was calculated for the whole sample (2-5-year-olds) and for each age group (2-, 3-, 4-, and 5-year olds). Children were classified by dietary pattern (omnivores, vegetarian/vegan) and dairy consumption. Multivariable linear regression assessed associations between iodine intake and sociodemographic and dietary factors. Analyses accounted for complex survey design.

**Results:**

Mean iodine intake was 148.9 µg/day (95% CI: 145.0, 152.8 µg/day; median: 143.5 µg/day; 25th, 75th percentiles: 120.2, 175.1 µg/day). Few 2-5-year-old children (1.1%) had iodine intakes below the EAR, while intakes in 18.4% of 2-year-old children and 14.9% of 3-year-old children exceeded the UL; none (0%) of 4–5‑year‑old children had iodine intakes that exceeded the UL. Intake declined with age (-7.5 µg/day; 95% CI: -10.5, -4.5 µg/day) and was higher in children from food-secure than food-insecure households (mean difference: 16.0 µg/day; 95% CI: 7.3, 24.8 µg/day). Dairy avoiders had lower intakes than consumers (mean difference: 25.7 µg/day; 95% CI: 11.1, 40.3 µg/day). No differences were observed by dietary pattern. Major iodine sources were dairy milk (34.8%), bread/bread rolls (24.4%), cereal-based dishes (4.8%), and yoghurt (4.5%).

**Conclusions:**

While inadequate intake was uncommon, excessive iodine intake affected over 1 in 7 2- and 3-year-old children. Iodine intake declined with age and was lower in children from food-insecure households and those avoiding dairy. Strategies are needed to address excess and inadequacy in vulnerable groups.

**Supplementary Information:**

The online version contains supplementary material available at 10.1007/s00394-026-04004-9.

## Introduction

Iodine is a trace mineral essential for the synthesis of thyroid hormones, which regulate metabolism, somatic growth, and neurodevelopment throughout life [1]. During early childhood, particularly the preschool years (2–5 years), insufficient iodine intake can impair thyroid hormone production, leading to iodine deficiency disorders such as goitre and hypothyroidism, with potential adverse effects on growth and neurodevelopment [2]. Even mild iodine deficiency has been associated with reduced cognitive function and impaired growth in young children [3–5]. Globally, iodine deficiency remains a significant public health concern and the leading cause of preventable intellectual disability [2], affecting nearly a third of the world’s population [6], including around 246 million children [7].

During the early 20th century, iodine deficiency was common in Australia [8]. The introduction of iodised salt in the 1920s, mid-20th century public health initiatives, and the widespread use of iodine-containing dairy sanitisers from the early 1960s eliminated iodine deficiency [9, 10]. However, the reduced use of iodine-based sanitisers in the 1970s, combined with a decline in the consumption of household iodised salt, led to the re-emergence of iodine deficiency [11, 12]. This prompted the introduction of mandatory iodine fortification in 2009, which required the use of iodised salt in all commercially produced bread, excluding organic varieties [13]. Post-fortification studies have reported improved iodine status in some population groups [14, 15], while others indicate that some preschool children may be at risk of excessive intakes [16, 17]. These variable findings highlight the need to investigate iodine intakes among Australian preschoolers.

Despite population-level improvements in iodine status in Australia, emerging dietary trends may present new challenges. The increasing popularity of plant-based diets, driven by environmental, health, and ethical concerns, may affect iodine intakes [18]. These diets vary in restrictiveness, with vegetarian diets excluding meat, poultry and fish, and vegan diets eliminating all animal-derived foods, including dairy [19]. This raises concerns, as plant-based dairy alternatives are naturally low in iodine and, unless fortified, contribute little to iodine intake [20]. Moreover, most plant foods are inherently low in iodine, as only a small fraction of soil iodine is water-soluble and available for uptake, even in iodine-rich soils [21]. Although seaweed is an iodine-rich exception, it is rarely consumed by young children in Australia [22]. Additionally, dietary guidelines for young children discourage adding salt to home-prepared and manufactured foods; as salt is the main vehicle for iodine fortification, this may further limit iodine intake [23]. Given the limited iodine content of plant-based foods, it is critical to assess whether preschool children following such dietary patterns are at increased risk of iodine deficiency.

Evidence on iodine intake adequacy among Australian preschool children is limited, and little is known about sociodemographic or dietary factors that may influence intake. Understanding whether iodine intake differs across sociodemographic groups and dietary patterns, and identifying sources of iodine is essential to evaluate if current Australian iodine fortification policies effectively reach all population groups and assess if subgroups of preschool children are at risk of inadequate or excessive intake. Therefore, this study aimed to assess the adequacy of iodine intake among a nationally representative sample of Australian preschoolers aged 2–5 years, and to examine sociodemographic and dietary factors, including plant-based dietary patterns, associated with iodine intake. Additionally, the study identified dietary sources of iodine in this population.

## Methods

### Study design and survey participants

This study is a secondary analysis of the 2011–2013 National Nutrition and Physical Activity Survey (NNPAS). The full NNPAS methods have been described previously [24]. The NNPAS was conducted by the Australian Bureau of Statistics (ABS) between May 2011 and June 2012 and was designed to provide nationally representative, cross-sectional data on dietary intake and physical activity. The survey employed a stratified, multistage, area-based sampling strategy and collected data from 9,500 private dwellings, from which one adult and one child (aged ≥ 2 years) were randomly chosen per household. This yielded a final sample of 12,153 participants, including 822 children aged 2–5 years, and a household response rate of 77% [24]. Separate person- and household-level weights were applied to account for the probability of selection, non-response, and seasonal variation, enabling inference to the total Australian population. Very remote areas and Aboriginal and Torres Strait Islander communities were excluded from the survey population [24].

Trained ABS interviewers conducted in-person interviews with the selected adult (aged ≥ 18 years; referred to herein as the adult-pair) per selected household to collect information on household-level and individual demographic and socioeconomic characteristics [24]. In addition, a nominated adult proxy provided information on behalf of the selected child aged 2–5 years. The proxy respondent provided data on the child’s demographic characteristics, and dietary intake which was collected using a 24-h dietary recall. Voluntary anthropometric measurements were collected from children by ABS interviewers [24]. The ABS conducted the survey under the Census and Statistics Act 1905, and written, informed consent was obtained from all adults and legal guardians of participating children [24].

### Dietary intakes

Dietary data (foods and beverages) and supplement intake data were collected through up to two non-consecutive 24-h recalls per child, reported by the nominated adult proxy. Recalls were conducted using the United States Department of Agriculture Automated Multiple-Pass Method, adapted by the ABS and Food Standards Australia New Zealand to reflect the Australian food supply [24]. All participants completed the first recall conducted in person via a Computer-Assisted Personal Interview. A subset of participants (*n* = 506, 62%) completed the second recall, which was scheduled for at least eight days after the first recall and conducted by telephone via a Computer-Assisted Telephone Interview [24]. The Automated Multiple-Pass Method uses structured series of prompts to help the respondent recall and report all foods consumed on the previous day. Food model booklets were used to support respondent recall and estimation of the quantities of foods and beverages consumed [24]. Participants also reported the name/brand, amount taken, and AUST-L identification number of all dietary supplements consumed in the previous 24 h. Dietary intake data (foods and beverages) were coded using the AUSNUT 2011–2013 food composition database, which was specifically developed by Food Standards Australia New Zealand for the NNPAS and contains nutrient information for 5740 foods, including iodine values that reflect both fortified and non‑fortified food products [25]. The AUST-L identification number, which represents the registration number for listed medicines on the Australian Register of Therapeutic Goods was used to match reported supplements against the register of dietary supplements sold in Australia and to accurately code their nutrient content [24, 25]. Daily intakes of energy and nutrients were estimated for each participant. Iodine intake was estimated as dietary iodine intake only, as well as with the contribution from supplements, defined as total iodine intake. Participants also answered two questions about food avoidance due to food allergies or intolerances (e.g., cow’s milk/dairy, eggs, gluten), or for cultural, religious or ethical reasons (e.g., meat, dairy, all animal products) [24]. Responses were used to define dietary patterns related to plant-based diets and dairy avoidance. Participants who reported avoiding animal tissue (i.e., all meat, poultry, and seafood, but not dairy or eggs) were classified as vegetarians, while those avoiding all animal products were classified as vegans. Participants who reported avoiding cow’s milk or dairy due to allergy/intolerance or for cultural, religious, or ethical reasons were classified as dairy avoiders; all others were classified as dairy consumers. In addition, participants reported whether salt was usually added to their meals during cooking or at the table (responses: very often, occasionally, rarely, not used, not known) and whether the salt was iodised (responses: iodised, not iodised, not known if iodised). Responses were used to classify participants as iodised salt users or non-users. Users included those who reported use of iodised salt at the table and/or during cooking. Non-users included those who used non-iodised salt, used salt with unknown iodisation status, did not use salt, or had unknown salt usage. Information on the quantity of discretionary salt used was not collected and reported iodine intakes do not include any iodine from discretionary salt.

### Sociodemographic factors

The survey obtained information on the child’s age and sex; the adult-pair’s country of birth, education level and employment status; and household data including family composition, household size, socioeconomic data, and food security [24]. Country of birth was classified into one of three categories: Australia, other main English-speaking countries (Canada, Ireland, New Zealand, South Africa, United Kingdom, and United States of America), or all other countries. Level of education was categorised as up to completion of final year of secondary school, completion of a trade certificate or diploma, or completion of an undergraduate degree or higher. Employment status was classified as employed (part-time or full-time employment) or unemployed/not in the workforce. Family composition and household size were described by family type (i.e., classified into families with two parents, single parent, or other structures) and the number of individuals residing in the household. The 2011 Index of Relative Socio-Economic Disadvantage, a component of the ABS Socio-Economic Indexes for Areas, was used to assess relative social and economic disadvantage for each participant based on the household postcode [24, 26]. This index summarises area-level socioeconomic characteristics using indicators such as income, education, employment, occupation, housing and family structure [26]. Based on this, participants were grouped into quintiles, ranging from the most disadvantaged (lowest quintile) to the least disadvantaged (highest quintile). Food security was assessed based on whether the household had run out of food in the last 12 months, with responses categorised as ‘Yes’ or ‘No’.

### Anthropometry and assessment of under-reporting

Children’s height and weight were measured using standard protocols by trained interviewers [24]. Body mass index was calculated as weight in kilograms divided by height in metres squared (kg/m²), and children were categorised as underweight, healthy weight, or overweight/obese according to the International Obesity Taskforce age- and sex-specific cut-offs [27]. Basal metabolic rate (BMR) was calculated using measured height and weight, using the Schofield equations [28]. This was completed in those children who had recorded measures of both height and weight (*n* = 625 of 822 children aged 2–5 years). Following this, the paediatric-adjusted Goldberg cut-off method [29] was used to identify potential under-reporting of energy intake. To do this, the ratio of energy intake (EI) to BMR (EI: BMR) was calculated for each child. The paediatric-adjusted Goldberg cut-off uses the original adult Goldberg cut-off 2 equation substituted with a child-specific physical activity level of 1.45 [30]. Of the 625 children with available height and weight data, 21 were identified as under-reporters and excluded from the analysis. Under-reporting could not be assessed for 158 children due to missing anthropometric data. As it is possible these children may have under-reported, but we were unable to determine this using the Goldberg method, we performed a sensitivity analysis whereby all analyses were repeated with additional exclusion of these children. Findings from this sensitivity analysis (*n* = 604) showed no appreciable difference to any of the reported findings (data not shown).

### Study sample

**Fig. 1:**
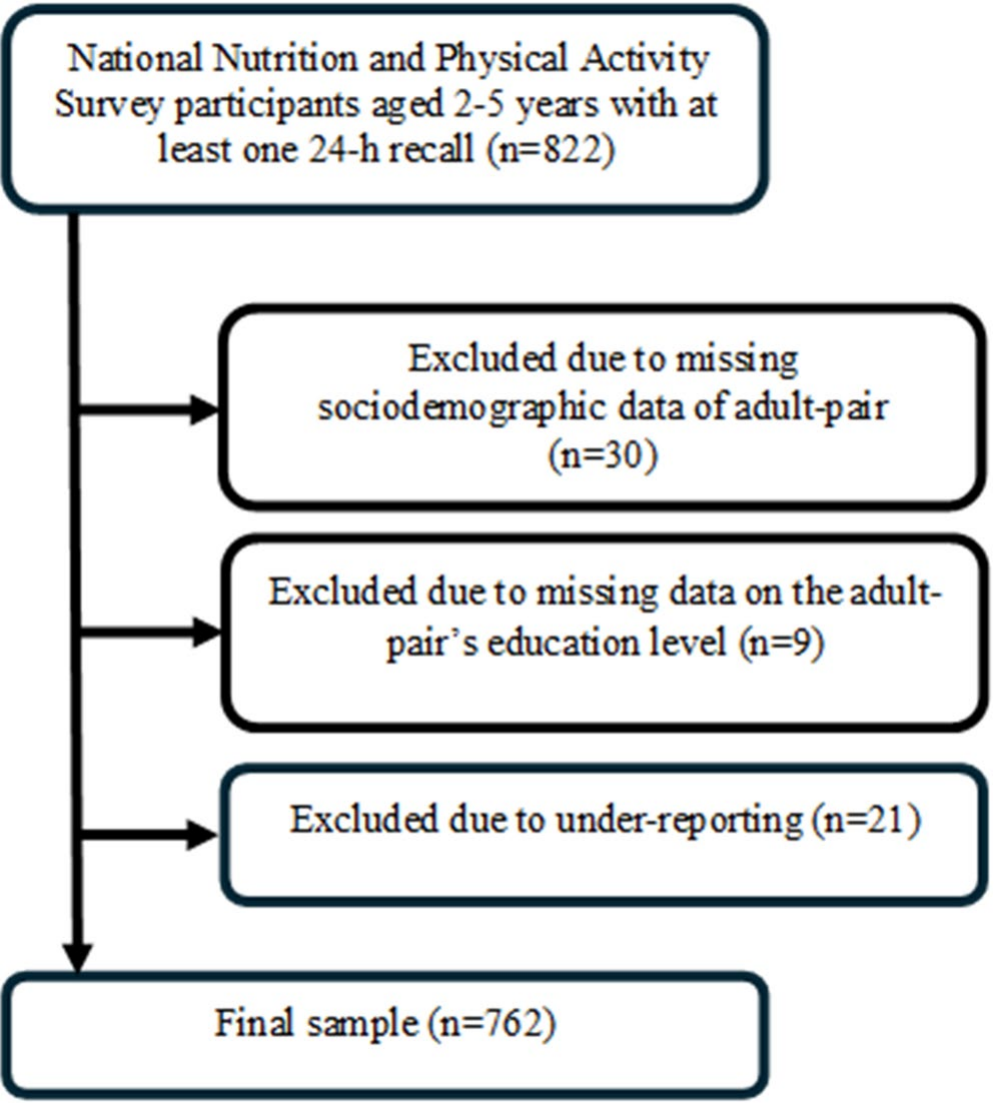
Flow diagram of eligible participants for the study sample

All children aged 2–5 years in the 2011–2013 NNPAS had at least one day of dietary recall data; therefore all were eligible for inclusion in the current study (*n* = 822). Children were excluded if they were the only household member recorded, resulting in no available sociodemographic data for their adult-pair (*n* = 30), had missing education data for the adult-pair (*n* = 9), or were identified as under-reporters of energy intake (*n* = 21), resulting in a final sample of 762 children (Fig. 1).

### Data analysis

Descriptive statistics were conducted to determine participant characteristics and usual intakes of energy, macronutrients, and dietary (i.e., iodine from food sources only) and total iodine (i.e., dietary iodine and iodine from supplements) for all children. Usual energy and nutrient intakes were estimated using the Multiple Source Method, which incorporated data from both children with only one 24-h recall (*n* = 287) and those with two recalls (*n* = 475), and modelled usual intake by adjusting for within‑person day‑to‑day variation in 24‑h recall data [31]. As only 30 children (4.3%) reported consuming iodine supplements, and we found no difference in iodine intake (dietary or total iodine) between supplement and non-supplement users (both p-values > 0.05), the remaining analyses were restricted to reporting on dietary iodine intake only (i.e., iodine from foods and beverages). A sensitivity analysis which used total iodine intake was completed and showed no appreciable change to any of the results (data not shown). The prevalence of inadequate dietary iodine intakes (i.e., proportion of children with usual intakes below the Australian/New Zealand Estimated Average Requirement (EAR) of 65 µg/day for 1-8-year-old children [32]) and excessive dietary intakes (i.e., proportion of children with usual intakes above the Australian/New Zealand Upper Level of Intake (UL) of 200 µg/day for 1-3- and 300 µg/day for 4-8-year-old children [32]) was estimated for the whole study sample, and by single-year age groups (i.e., 2‑, 3‑, 4‑, and 5‑year‑olds). Contributions of iodine from food groups were assessed using the AUSNUT 2011–2013 Food Classification System, which groups food items into major, sub-major, and minor groups [25]. The population proportion method was applied to estimate the percentage contribution of each food group to overall iodine intake [33]. Differences in usual dietary iodine intake between participants who were classified as dairy consumers and dairy avoiders, and omnivores versus vegetarians/vegans were examined using linear regression analysis with adjustment for energy intake and discretionary iodised salt use. Vegetarians/vegans were combined for analysis due to the very low numbers in each group (vegetarians: *n* = 10, vegans: *n* = 2). Linear regression was used to examine sociodemographic factors associated with usual dietary iodine intake. The base regression models were adjusted for energy intake and discretionary iodised salt use. Potential correlates included were age (continuous variable) and sex of child; adult-pair’s country of birth, education level, and employment status; and household area-level disadvantage quintile, food security status, household type (all categorical variables), and household size (continuous variable). Variables with p-values < 0.20 in base regression analyses (i.e., child sex and age; and household size and food security) were simultaneously included in multivariable regression models to assess independent associations. The distribution and variance of residuals from all regression models were examined to assess model assumptions. All statistical analyses were performed using Stata BE (version 18; StataCorp LLC, College Station, TX, USA). Survey weights were applied to ensure estimates were representative of the Australian population, and jackknife replicate weights were used to calculate standard errors that accounted for the survey’s complex, clustered sampling design. Results were considered statistically significant at *p* < 0.05.

## Results

### Participant characteristics and nutrient intakes

The mean (SD) age of preschool children in the study sample (*n* = 762) was 3.5 (1.1) years with a balanced distribution across age and sex categories (Table 1). Most of the children were of a healthy weight (70.6%). Salt use during cooking or at the table was reported for just over half of the children (53.2%), with iodised salt use (25.5%) more common than non-iodised salt use (20.4%). Few children used iodine supplements (4.3%). Most children lived in households where the adult-pair was Australian born (72.5%) and employed (67.9%). Educational attainment of the adult-pair was relatively evenly distributed, with approximately one third (34.2%) having a tertiary education. Most households were food secure (93.3%), with two-parent families (79.3%) and four-person households (41.0%) being most common. The mean (SD) household size was 4.2 (1.0) persons. Socioeconomic disadvantage of households was relatively evenly represented (Table 1). Mean usual energy intake among preschool children was 6144 (95% CI: 6046, 6243) kJ/day (1 kcal = 4.186 kJ). Mean usual protein, fat and carbohydrate intakes were 58.3 (95% CI: 57.0, 59.5) g/day, 51.2 (95% CI: 50.0, 52.4) g/day, and 186.8 (95% CI: 183.3, 190.3) g/day, respectively.

**Table 1:** Characteristics of participants aged 2–5 years from the 2011–2013 Australian National Nutrition and Physical Activity Survey (*n* = 762)

	*n* ^a^	%
Age of child (years)		
2	224	24.8
3	204	25.6
4	195	27.4
5	139	22.3
Sex of child		
Male	369	50.2
Female	393	49.8
Body mass index categories ^b^		
Underweight	29	5.4
Healthy weight	433	70.6
Overweight/obese	142	24.0
Salt users		
Yes, iodised	212	25.5
Yes, not iodised	137	20.4
Yes, iodisation unknown	47	7.3
No	361	46.5
Unknown if salt used	5	0.2
Iodine supplement users		
Yes	30	4.3
No	732	95.7
Country of birth of adult-pair		
Australia	557	72.5
Main English-speaking countries ^c^	66	9.2
Other countries	139	18.3
Level of education of adult-pair		
Undergraduate university degree or higher	269	34.2
Trade certificate or diploma	289	39.8
Up to completion of final year of secondary school	204	26.0
Employment status of adult-pair		
Employed	537	67.9
Unemployed/not in the workforce	225	32.1
Index of Relative Socio-Economic Disadvantage of household ^d^		
Lowest quintile	143	20.8
Second quintile	150	18.8
Third quintile	156	20.9
Fourth quintile	138	17.2
Fifth quintile	175	22.4
Food security of household		
Ran out of food in last 12 months	51	6.7
Did not run out of food in last 12 months	711	93.3
Household type		
Two-parent family	597	79.3
Single-parent family	98	13.4
Other households	67	7.3
Household size		
Two-person household	41	3.2
Three-person household	193	17.1
Four-person household	341	41.0
Five-person household	146	28.9
Six-or-more-person household	41	9.8

^a^ n (% weighted)

^b^ *n* = 158 children with missing height and/or weight; Body mass index categories are based on the following half-year cut-off points for individual age and sex. Males: 2 years: <15.14 underweight, 15.14–18.41 healthy weight, > 18.41 overweight/obese; 2.5 years: <14.92 underweight, 14.92–18.13 healthy weight, > 18.13 overweight/obese; 3 years: <14.74 underweight, 14.74–17.89 healthy weight, > 17.89 overweight/obese; 3.5 years: <14.47 underweight, 14.47–17.69 healthy weight, > 17.69 overweight/obese; 4 years: <14.43 underweight, 14.43–17.55 healthy weight, > 17.55 overweight/obese; 4.5 years: <14.31 underweight, 14.31–17.47 healthy weight, > 17.47 overweight/obese; 5 years: <14.21 underweight, 14.21–17.42 healthy weight, > 17.42 overweight/obese; 5.5 years: <14.13 underweight, 14.13–17.45 healthy weight, > 17.45 overweight/obese. Females: 2 years: <14.83 underweight, 14.83–18.02 healthy weight, > 18.02 overweight/obese; 2.5 years: <14.63 underweight, 14.63–17.76 healthy weight, > 17.76 overweight/obese; 3 years: <14.47 underweight, 14.47–17.56 healthy weight, > 17.56 overweight/obese; 3.5 years: <14.32 underweight, 14.32–17.40 healthy weight, > 17.40 overweight/obese; 4 years: <14.19 underweight, 14.19–17.28 healthy weight, > 17.28 overweight/obese; 4.5 years: <14.06 underweight, 14.06–17.19 healthy weight, > 17.19 overweight/obese; 5 years: <13.94 underweight, 13.94–17.15 healthy weight, > 17.15 overweight/obese; 5.5 years: <13.86 underweight, 13.86–17.20 healthy weight, > 17.20 overweight/obese

^c^ Canada, New Zealand, South Africa, Ireland, United Kingdom, United States of America

^d^ Socio-Economic Indexes for Areas (SEIFA) 2011

### Iodine intake and associations with sociodemographic factors

The mean usual dietary iodine intake was 148.9 (95% CI: 145.0, 152.8) µg/day (median: 143.5 µg/day; 25th, 75th percentiles: 120.2, 175.1 µg/day). The mean usual total iodine intake was 150.1 (95% CI: 146.3, 153.9) µg/day (median: 145.6 µg/day; 25th, 75th percentiles: 120.8, 175.6 µg/day). After adjustment for energy intake and iodised salt use, no significant differences were observed between supplement users and non-users for usual dietary iodine intake (mean difference: -9.5 µg/day; 95% CI: -21.5, 2.6 µg/day; *p* = 0.121) or usual total iodine intake (mean difference: 10.5 µg/day; 95% CI: -4.6, 25.7 µg/day; *p* = 0.169). Table 2 shows the proportion of children with dietary iodine intake below the EAR and those exceeding the UL, overall and by age group. Few preschool children had dietary iodine intake below the EAR (1.1%; 95% CI: 0.4, 2.8%), while 8.4% (95% CI: 5.9, 11.8%) exceeded the UL. Excessive intake was most common among 2-year-olds (18.4%; 95% CI: 12.0, 27.3%) and 3-year-olds (14.9%; 95% CI: 8.8, 24.2%).

**Table 2:** Prevalence of inadequate and excessive dietary iodine intake in Australian preschool children (*n* = 762) by age group

Age	% below EAR (95% CI) ^a^	% above UL (95% CI) ^b^
All ages (*n* = 762)	1.1 (0.4, 2.8)	8.4 (5.9, 11.8)
2 years (*n* = 224)	0.8 (0.1, 5.8)	18.4 (12.0, 27.3)
3 years (*n* = 204)	1.9 (0.4, 8.3)	14.9 (8.8, 24.2)
4 years (*n* = 195)	1.5 (0.3, 6.9)	0.0 (0.0, 0.0)
5 years (*n* = 139)	0.0 (0.0, 0.0)	0.0 (0.0, 0.0)

EAR: Estimated Average Requirement; UL: Upper Level of Intake

^a^ Inadequate intake defined as < 65 µg/day (Australian/New Zealand EAR for 1–8 years) [32]; ^b^ Excessive intake defined as > 200 µg/day for 2–3 years (Australian/New Zealand UL for children aged 1–3 years) and > 300 µg/day for 4–5 years (Australian/New Zealand UL for children aged 4–8 years) [32]

In the base regression models adjusted for energy intake and iodised salt use, child age and food security were significantly associated with dietary iodine intake among Australian preschoolers (Table 3). In the multivariable regression model both associations remained significant (Table 4). Mean dietary iodine intake decreased by 7.5 µg/day for each additional year of age (95% CI: -10.5, -4.5 µg/day; *p* < 0.001), and children from households that had not run out of food in the last 12 months had mean dietary iodine intakes that were 16.0 µg/day higher than those from households that had run out of food in the last 12 months (95% CI: 7.3, 24.8 µg/day; *p* = 0.001).

**Table 3:** Associations between sociodemographic characteristics and usual dietary iodine intakes adjusted for energy intake and iodised salt use among Australian preschool children (*n* = 762)

	Mean iodine intake (µg/day)	SE	B co-efficient	95% CI	*p* value
Age of child (years)			-7.2	-10.0, -4.2	< 0.001
2	159.6	2.7			
3	152.3	1.8			
4	145.1	1.8			
5	137.9	2.7			
Sex of child					0.175
Male	146.4	2.7			
Female	151.5	2.3	5.1	-2.3, 12.5	
Country of birth of adult-pair					0.325
Australia	147.4	1.8			
Main English-speaking countries ^a^	155.6	5.7	8.2	-4.2, 20.6	
Other countries	151.6	4.4	4.3	-4.7, 13.3	
Level of education of adult-pair					0.229
Undergraduate university degree or higher	151.8	2.8			
Trade certificate or diploma	148.5	3.7	-3.3	-13.5, 6.9	
Up to completion of final year of secondary school	145.7	2.4	-6.1	-13.3, 1.0	
Employment status of adult-pair					0.551
Employed	148.2	2.2			
Unemployed/not in the workforce	150.4	2.8	2.2	-5.1, 9.6	
Index of Relative Socio-Economic Disadvantage of household ^b^					0.312
Lowest quintile	147.0	3.8			
Second quintile	142.8	4.2	-4.2	-16.6, 8.1	
Third quintile	149.5	3.0	2.5	-6.4, 11.4	
Fourth quintile	155.7	4.3	8.6	-3.3, 20.6	
Fifth quintile	150.0	4.1	3.0	-7.3, 13.4	
Food security of household					0.046
Ran out of food in last 12 months	139.9	4.6			
Did not run out of food in last 12 months	149.6	1.7	9.7	0.2, 19.2	
Household type					0.237
Two-parent family	148.4	1.8			
Single-parent family	146.9	4.6	-1.5	-11.3, 8.3	
Other households	158.5	6.0	10.2	-2.3, 22.6	
Household size			-3.8	-7.6, 0.1	0.054
Two-person household	157.4	4.5			
Three-person household	153.6	2.8			
Four-person household	149.8	1.7			
Five-person household	146.1	2.3			
Six-or-more-person household	142.3	3.9			

^a^ Canada, New Zealand, South Africa, Ireland, United Kingdom, United States of America

^b^ Socio-Economic Indexes for Areas (SEIFA) 2011

**Table 4:** Multivariable model of correlates of usual dietary iodine intakes adjusted for energy intake and iodised salt use in Australian preschool children (*n* = 762)

	B co-efficient ^a^	95% CI	*p* value
Age of child (years)	-7.5	-10.5, -4.5	< 0.001
Sex of child			0.080
Male			
Female	6.3	-0.8, 13.4	
Food security of household			0.001
Ran out of food in last 12 months			
Did not run out of food in last 12 months	16.0	7.3, 24.8	
Household size (persons per household)	-3.4	-7.0, 0.3	0.068

^a^ *B* coefficient represents difference in iodine intake adjusted for energy intake, iodised salt use, and all variables listed in the table

### Associations between iodine intake and dietary factors

Of the 762 preschool children, 728 (96.4%) consumed dairy products and 34 (3.6%) avoided them. After adjustment for energy intake and iodised salt use, dairy consumers had significantly higher mean usual dietary iodine intake (149.8 µg/day; 95% CI: 146.4, 153.2 µg/day) compared to dairy avoiders (124.1 µg/day; 95% CI: 109.5, 138.7 µg/day), with a mean difference in intake of 25.7 µg/day (95% CI: 11.1, 40.3 µg/day; *p* = 0.001). Seven hundred and fifty (98.4%) children were classified as omnivores, while 12 (1.6%) were classified as vegetarian (*n* = 10) or vegan (*n* = 2). After adjustment for energy intake and iodised salt use, the mean dietary iodine intake was 160.3 (95% CI: 106.9, 213.6) µg/day among vegetarian/vegan children (*n* = 12) and 148.8 (95% CI: 145.5, 152.2) µg/day among omnivorous children, with an adjusted mean difference of 11.4 µg/day (95% CI: -42.3, 65.1 µg/day; *p* = 0.672).

### Sources of iodine intake

**Fig. 2:**
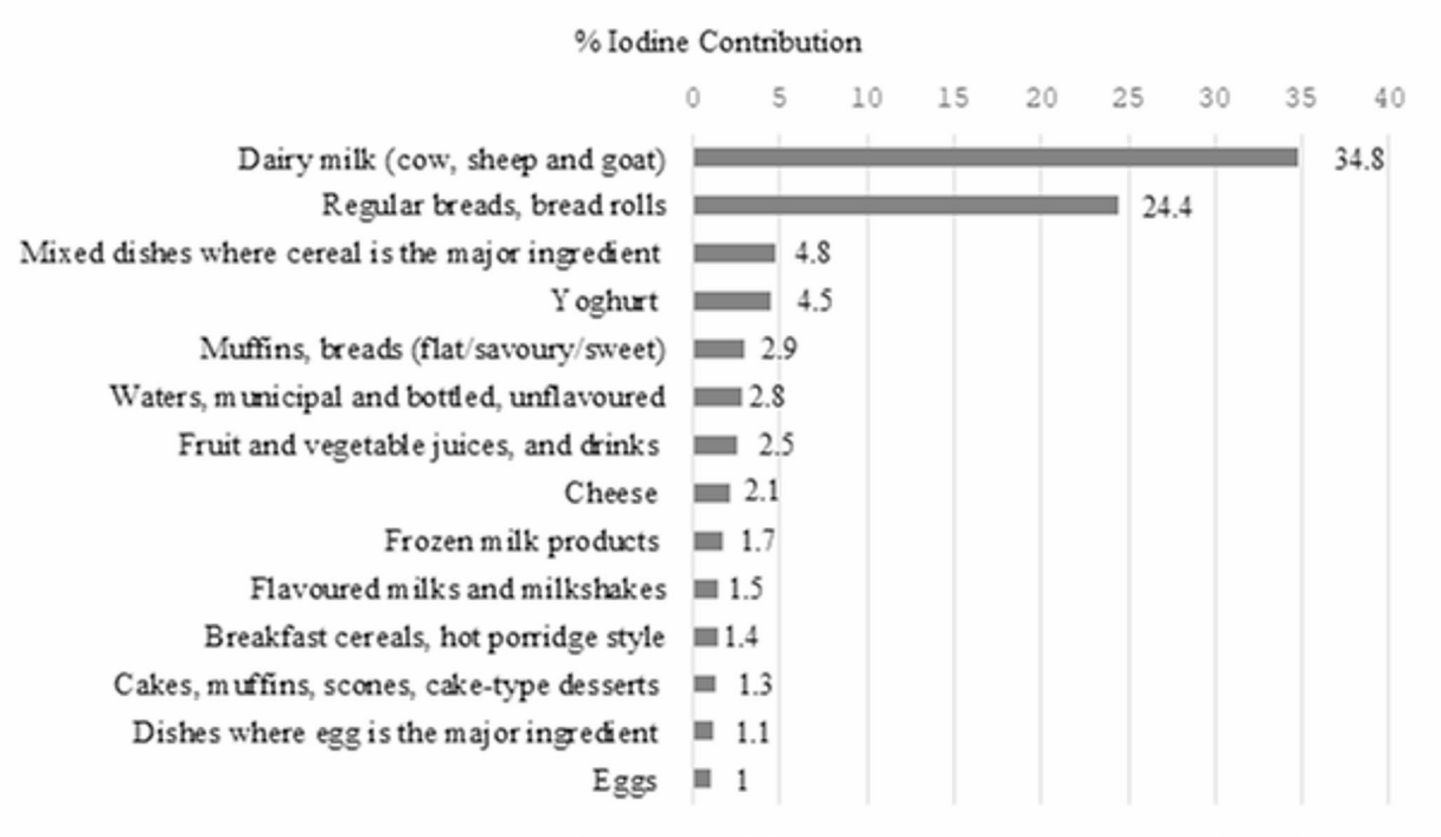
Major dietary iodine sources contributing ≥ 1% of total dietary iodine intake among Australian preschool children aged 2–5 years (*n* = 762)

Dietary sources of iodine contributing ≥ 1% of total dietary intake among Australian preschool children are illustrated in Fig. 2. Dairy milk was the largest contributor, accounting for 34.8% of total dietary iodine intake, followed by regular bread and bread rolls (24.4%), mixed dishes where cereal was the major ingredient (4.8%; e.g., pasta‑ or rice-based dishes), and yoghurt (4.5%). Of the remaining 10 sources, each contributed < 3% to total dietary iodine intake. A full list of all iodine sources, including those contributing < 1% to total intake, is provided in Online Supplementary Table 1.

## Discussion

This study examined adequacy of iodine intake and was the first to identify potential sociodemographic and dietary factors associated with iodine intake among a nationally representative sample of Australian preschool children aged 2–5 years. Most children had usual iodine intake that met or exceeded the EAR, with excessive intake observed among approximately 15% of children aged 2–3 years. Age was negatively associated, and food security was positively associated with iodine intake. Dairy avoiders had significantly lower iodine intakes than dairy consumers. Iodine intakes did not differ between omnivores and vegetarians/vegans, or between iodine supplement users and non-users, however, these subgroup analyses were based on very small numbers of children, limiting statistical power and precision of estimates. Major iodine sources were milk, bread, and cereal-based dishes.

Inadequate iodine intake was rare across all age groups in our study, while excessive iodine intake was relatively common in 2-3-year-old children. These results align with those reported in some [17, 34, 35] but not all [36, 37] countries reflecting variation in dietary patterns, soil iodine levels, and public health policies. In Australia, all commercially produced breads, except organic varieties, are fortified with iodine [13]. While Australia’s current fortification policy has effectively reduced iodine inadequacy, it may be contributing to excess intakes among some groups, particularly younger preschool children. This likely reflects the widespread consumption of iodine-fortified bread combined with the relatively lower UL for 2-3-year-olds compared to older age groups. Excessive iodine exposure in this age group can impair thyroid function and lead to both hypothyroidism and hyperthyroidism, which adversely affect growth and neurodevelopment [38]. Ongoing monitoring is needed to ensure fortification policies are appropriately balanced to minimise the risk of both deficiency and excess.

As preschoolers depend on caregivers for their nutrition [39], family sociodemographic circumstances may be important determinants of health-related behaviours, such as dietary intake. In this study, among the factors examined, only child’s age and food security were significantly associated with iodine intakes in the multivariable analysis. Iodine intake declined with age, consistent with previous research in Australia [40] and Belgium [35], with both studies reporting lower iodine status or intake in older preschool children. The Australian study found a decline in urinary iodine concentration of ≈ 15 µg/L between ages 2 and 5 years [40], and the Belgian study reported a decrease in iodine intake of ≈ 10 µg/day between ages 2.5-4 and 4-6.5 years [35]. These consistent findings likely reflect age-related changes in dietary behaviour, as children’s diets transition to include more table food and fewer dairy products, particularly milk [41], which are major contributors to iodine intake in this age group [42]. In contrast, no significant age-related changes in iodine intake were found in Chinese children aged 3–6 years [37] and Korean children aged 2–7 years [36]. However, the main source of iodine in these countries is seaweed, a rich natural source of iodine that is consumed regularly in many Asian cultures [43], with intake remaining relatively stable throughout early childhood [36].

Evidence on food security and iodine intake in children is limited, with few studies having examined this relationship. In the present study, children living in food-insecure households had significantly lower iodine intakes than children from food-secure households. This contrasts with findings from Colombia [44], where no significant association was observed between food security and iodine status after multivariable adjustment, likely reflecting the widespread use of iodised salt in the population and its heavy fortification, which minimises differences between groups. Nevertheless, food insecurity may increase the risk of inadequate iodine intake among Australian preschoolers due to reduced access to iodine-rich foods such as dairy milk and bread, highlighting the need for policy and practice to improve access and address the underlying causes of food insecurity [45].

Other sociodemographic factors, such as child’s sex; the adult-pair’s level of education, employment status, and country of birth; and household type, size, and socioeconomic status were not associated with iodine intake in the present study. Similarly, previous studies reported no significant sex differences in iodine status among children [36, 40, 46, 47]. In contrast, evidence from Finland [34], China [37], and Spain [48] shows that children of more educated parents have significantly higher iodine intakes or status. In the present study, education level was reported for the designated adult-pair, who was not necessarily the child’s parent [24], potentially limiting the ability to capture true parental education influences. The associations between parental country of birth and employment status, and household type, size, and socioeconomic status and iodine intake have not previously been explored in preschool children.

In the present study, dairy products, particularly milk and yogurt, as well as bread and cereal-based products, were identified as the primary dietary iodine sources in Australian preschool children. Dairy milk was the largest contributor (≈ 35% of total dietary iodine intake), which aligns with findings from other international studies, although the magnitude of its contribution varies widely across populations (13%-65%) [35, 46, 47, 49]. Bread and cereal-based products were found to contribute nearly one third to preschoolers’ dietary iodine intake in the present study. Similarly, a Belgian study identified bread as a major iodine source, though it only contributed 8% of total iodine intake [35]. The significantly higher contribution observed in Australia likely reflects mandatory fortification with iodine, compared to Belgium’s voluntary program at lower iodisation levels.

Only a small proportion of preschool children were identified as vegetarian/vegan in the present study (1.6%), and their iodine intake did not differ significantly from that of omnivores. Similar findings were reported in a small Finnish study [50]. In contrast, two larger studies reported significantly lower median iodine intakes among vegetarian and vegan preschoolers compared to omnivores, with differences of approximately 14 µg/day in Germany [51] and 20 µg/day in the Czech Republic [52]. In Germany, this difference persisted even after accounting for supplement use [51]. The low number of vegetarian and vegan children in the present study likely reflects the timing of data collection for the 2011–2013 NNPAS, which preceded the rise in popularity of plant-based diets in Australia. While current prevalence data for preschool children are unavailable, recent national estimates indicate that approximately 5.3% of Australians follow a vegetarian or vegan diet [53]. Although the NNPAS dataset is over a decade old, these recent statistics suggest that the proportion of Australians adhering to vegetarian or vegan diets has remained relatively stable, supporting the continued relevance of the dataset. Additionally, the widespread consumption of fortified bread, a key iodine source for all dietary groups in Australia [54], may have contributed to the lack of observed differences in iodine intake between vegetarians/vegans and omnivores in this study.

Despite the relatively small number of dairy avoiders in the sample (*n* = 34), dairy consumers had significantly higher mean iodine intakes compared to dairy avoiders. Given that dairy milk contributed the largest share of iodine intake (≈ 35%), its exclusion likely reduced intake. Most plant-based alternatives lack iodine-fortification in Australia [55], which may explain the significantly lower iodine intakes observed among dairy avoiders. Although research in young children is limited, evidence from Australian [55, 56] and international adult populations [57, 58] similarly shows that avoiding dairy or substituting it with plant-based alternatives is associated with lower iodine intake and status. These findings highlight that, despite the consumption of iodine-fortified bread, avoiding dairy may potentially increase the risk of inadequate iodine intake in Australian preschoolers.

This study has several strengths. A key strength is the use of dietary data from the 2011–2013 NNPAS, a large, nationally representative survey that includes Australian children aged 2 years and older. The use of a validated dietary assessment method and collection of a second 24-h recall for most children, at least eight days later, enabled adjustment for within-person variation and determination of usual iodine intake. The AUSNUT 2011–2013 food composition database, which was developed specifically for this survey [25], further strengthened the accuracy of food coding and nutrient estimations. Additionally, the exclusion of energy under-reporters addressed a well-recognised limitation of self-reported dietary intakes and improved the reliability of the intake estimates [59]. The study also used robust statistical methods. The study limitations should also be noted. The amount of discretionary salt used was not directly measured in the 2011–2013 NNPAS, and therefore the contribution of iodised salt to iodine intake could not be determined. Reported iodine intakes may therefore underestimate true iodine intake, and the proportion of children exceeding the UL may be even higher among those who reported using discretionary salt than estimated in the current analysis. The small number of vegetarian/vegan children and iodine supplement users also limited the precision of these subgroup analyses. In addition, in the absence of contemporary nationally representative dietary data, ongoing monitoring of iodine intake among preschool children is warranted as new dietary survey datasets become available and the Australian/New Zealand Nutrient Reference Values for iodine are updated.

In summary, this study examined iodine intake and potential socioeconomic and dietary correlates among a nationally representative sample of Australian preschool children aged 2–5 years. The proportion of children with usual iodine intakes below the EAR was low, while at least 1 in 7 children aged 2–3 years exceeded the UL for iodine intake. Age and food security were the only sociodemographic factors associated with dietary iodine intakes, with age negatively and food security positively associated. Among dietary factors, dairy avoidance was associated with significantly lower iodine intakes compared to dairy consumption. No differences were found between vegetarians/vegans and omnivores, or iodine supplement users and non-users. Dairy products, specifically milk, bread, and cereal-based products were the main dietary iodine sources. Future research should focus on strategies to minimise excessive iodine intake among younger preschool children and investigate vulnerable groups such as dairy avoiders and those experiencing food insecurity. Additionally, fortification policies should be reviewed to ensure they meet the nutritional needs of all age groups without contributing to excessive intakes in younger children.

## Electronic Supplementary Material

Below is the link to the electronic supplementary material.

Supplementary Material 1

## Data Availability

Data used in the manuscript come from the 2011-2013 National Nutrition and Physical Activity Survey. Access to microdata from this study is available via the Australian Bureau of Statistics. For further information please see https://www.abs.gov.au/statistics/microdata-tablebuilder/available-microdata-tablebuilder/australian-health-survey-nutrition-and-physical-activity.
