# Supplementary material for: Iodine intake and its association with sociodemographic and dietary factors in Australian preschool children
Source: Eur J Nutr. 2026 Jun 19;65(5):165. doi: 10.1007/s00394-026-04004-9 (PMC13282298; doi:10.1007/s00394-026-04004-9)
Supplement: Supplementary file 1 — Supplementary Material 1 [file 394_2026_4004_MOESM1_ESM.docx]

**Iodine intake and its association with sociodemographic and dietary factors in Australian preschool children**

***European Journal of Nutrition***

Marion E. Rogerson, Carley A. Grimes, Ewa A. Szymlek-Gay

**Correspondence:** Ewa A. Szymlek-Gay. Email: [ewa.szymlekgay@deakin.edu.au](mailto:ewa.szymlekgay@deakin.edu.au)

**Online Supplementary Table 1**. Contribution of iodine from food groups [1] in the diets of preschool children aged 2-5 years (n=762)

| Food Group Code | Food Group and Sub-Group Description | % Iodine Contribution | 95% CI |
| --- | --- | --- | --- |
| 11 | **Non-alcoholic beverages** | 6.7 | 6.0, 7.3 |
| 111 | Tea | 0.04 | 0.0002, 0.08 |
| 112 | Coffee and coffee substitutes | 0.07 | 0.00 ^a^, 0.16 |
| 113 | Fruit and vegetable juices, and drinks | 2.5 | 2.0, 3.0 |
| 114 | Cordials | 0.3 | 0.2, 0.4 |
| 115 | Soft drinks, and flavoured mineral waters | 0.2 | 0.1, 0.3 |
| 116 | Electrolyte, energy and fortified drinks | 0.02 | 0.00 ^a^, 0.06 |
| 117 | Waters, municipal and bottled, unflavoured | 2.8 | 2.6, 3.1 |
| 118 | Other beverage flavourings and prepared beverages | 0.7 | 0.3, 1.1 |
| 12 | **Cereals and cereal products** | 29.7 | 27.8, 31.6 |
| 121 | Flours and other cereal grains and starches | 0.4 | 0.3, 0.5 |
| 122 | Regular breads, and bread rolls (plain/unfilled/untopped varieties) | 24.4 | 22.7, 26.1 |
| 123 | English-style muffins, flat breads, and savoury and sweet breads | 2.9 | 2.2, 3.6 |
| 124 | Pasta and pasta products (without sauce) | 0.2 | 0.1, 0.2 |
| 125 | Breakfast cereals, ready to eat | 0.4 | 0.3, 0.5 |
| 126 | Breakfast cereals, hot porridge style | 1.4 | 0.7, 2.1 |
| 13 | **Cereal based products and dishes** | 7.9 | 6.6, 9.1 |
| 131 | Sweet biscuits | 0.7 | 0.5, 0.9 |
| 132 | Savoury biscuits | 0.2 | 0.2, 0.3 |
| 133 | Cakes, muffins, scones, cake-type desserts | 1.3 | 1.0, 1.6 |
| 134 | Pastries | 0.5 | 0.2, 0.8 |
| 135 | Mixed dishes where cereal is the major ingredient | 4.8 | 3.6, 6.0 |
| 136 | Batter-based products | 0.4 | 0.1, 0.8 |
| 14 | **Fats and oils** | 0.1 | 0.1, 0.2 |
| 141 | Butters | 0.08 | 0.06, 0.09 |
| 142 | Dairy blends | 0.010 | 0.002, 0.016 |
| 143 | Margarine and table spreads | 0.04 | 0.03, 0.05 |
| 144 | Plant oils | 0 |  |
| 145 | Other fats | 0 |  |
| 146 | Unspecified fats | 0.01 | 0.0004, 0.01 |
| 15 | **Fish and seafood products and dishes** | 1.8 | 1.0, 2.6 |
| 151 | Fin fish (excluding commercially sterile) | 0.8 | 0.3, 1.3 |
| 152 | Crustacea and molluscs (excluding commercially sterile) | 0.05 | 0.00 ^a^, 0.10 |
| 153 | Other sea and freshwater foods | 0 |  |
| 154 | Packed (commercially sterile) fish and seafood | 0.10 | 0.02, 0.19 |
| 155 | Fish and seafood products (homemade and takeaway) | 0.8 | 0.1, 1.3 |
| 156 | Mixed dishes with fish or seafood as the major component | 0.1 | 0.00 ^a^, 0.2 |
| 16 | **Fruit products and dishes** | 0.6 | 0.6, 0.7 |
| 161 | Pome fruit | 0.2 | 0.2, 0.3 |
| 162 | Berry fruit | 0.05 | 0.02, 0.07 |
| 163 | Citrus fruit | 0.05 | 0.04, 0.07 |
| 164 | Stone fruit | 0.07 | 0.03, 0.10 |
| 165 | Tropical and subtropical fruit | 0.11 | 0.09, 0.13 |
| 166 | Other fruit | 0.07 | 0.06, 0.09 |
| 167 | Mixtures of two or more groups of fruit | 0.02 | 0.01, 0.04 |
| 168 | Dried fruit, preserved fruit | 0.03 | 0.01, 0.04 |
| 169 | Mixed dishes where fruit is the major component | 0.003 | 0.00 ^a^, 0.010 |
| 17 | **Egg products and dishes** | 2.2 | 1.3, 3.0 |
| 171 | Eggs | 1.0 | 0.5, 1.5 |
| 172 | Dishes where egg is the major ingredient | 1.1 | 0.5, 1.7 |
| 18 | **Meat, poultry and game products and dishes** | 1.8 | 1.4, 2.2 |
| 181 | Beef, sheep and pork, unprocessed | 0.07 | 0.03, 0.12 |
| 182 | Mammalian game meats | 0.001 | 0.00 ^a^, 0.005 |
| 183 | Poultry and feathered game | 0.2 | 0.1, 0.2 |
| 184 | Organ meats and offal, products and dishes | 0 |  |
| 185 | Sausages, frankfurts and saveloys | 0.2 | 0.1, 0.2 |
| 186 | Processed meat | 0.2 | 0.2, 0.3 |
| 187 | Mixed dishes where beef, sheep, pork or mammalian game is the major component | 0.3 | 0.1, 0.5 |
| 188 | Mixed dishes where sausage, bacon, ham or other processed meat is the major component | 0.0004 | 0.00 ^a^, 0.0011 |
| 189 | Mixed dishes where poultry or feathered game is the major component | 0.8 | 0.5, 1.1 |
| 19 | **Milk products and dishes** | 45.3 | 42.9, 47.6 |
| 191 | Dairy milk (cow, sheep and goat) | 34.8 | 32.2, 37.4 |
| 192 | Yoghurt | 4.5 | 3.7, 5.3 |
| 193 | Cream | 0.09 | 0.00 ^a^, 0.19 |
| 194 | Cheese | 2.1 | 1.8, 2.5 |
| 195 | Frozen milk products | 1.7 | 1.2, 2.1 |
| 196 | Custards | 0.2 | 0.1, 0.4 |
| 197 | Other dishes where milk or a milk product is the major component | 0.4 | 0.2, 0.6 |
| 198 | Flavoured milks and milkshakes | 1.5 | 0.8, 2.7 |
| 20 | **Dairy & meat substitutes** | 0.13 | 0.07, 0.19 |
| 201 | Dairy milk substitutes, unflavoured | 0.09 | 0.04, 0.13 |
| 202 | Dairy milk substitutes, flavoured | 0.003 | 0.00 ^a^, 0.008 |
| 203 | Cheese substitute | 0 |  |
| 204 | Soy-based ice confection | 0.0004 | 0.0004, 0.001 |
| 205 | Soy-based yoghurts | 0.008 | 0.00 ^a^, 0.018 |
| 206 | Meat substitutes | 0.031 | 0.005, 0.058 |
| 207 | Dishes where meat substitutes are the major component | 0 |  |
| 21 | **Soup** | 0.4 | 0.1, 0.6 |
| 211 | Soup, homemade from basic ingredients | 0.3 | 0.1, 0.6 |
| 212 | Dry soup mix | 0 |  |
| 213 | Soup, prepared from dry soup mix | 0.026 | 0.00 ^a^, 0.053 |
| 214 | Canned condensed soup (unprepared) | 0 |  |
| 215 | Soup, commercially sterile, prepared from condensed or sold ready to heat | 0.012 | 0.00 ^a^, 0.026 |
| 216 | Soup, not commercially sterile, purchased ready to eat | 0.0002 | 0.00 ^a^, 0.0007 |
| 22 | **Seed and nut products and dishes** | 0.06 | 0.04, 0.07 |
| 221 | Seeds and seed products | 0.003 | 0.00 ^a^, 0.008 |
| 222 | Nuts and nut products | 0.06 | 0.04, 0.07 |
| 23 | **Savoury sauces and condiments** | 0.2 | 0.2, 0.3 |
| 231 | Gravies and savoury sauces | 0.1 | 0.1, 0.2 |
| 232 | Pickles, chutneys and relishes | 0.01 | 0.00 ^a^, 0.01 |
| 233 | Salad dressings | 0.07 | 0.01, 0.13 |
| 234 | Stuffings | 0 |  |
| 235 | Dips | 0.027 | 0.00 ^a^, 0.056 |
| 24 | **Vegetable products and dishes** | 1.1 | 0.8, 1.4 |
| 241 | Potatoes | 0.3 | 0.3, 0.4 |
| 242 | Cabbage, cauliflower and similar brassica vegetables | 0.09 | 0.04, 0.15 |
| 243 | Carrot and similar root vegetables | 0.04 | 0.03, 0.05 |
| 244 | Leaf and stalk vegetables | 0.1 | 0.00 ^a^, 0.4 |
| 245 | Peas and beans | 0.017 | 0.005, 0.029 |
| 246 | Tomato and tomato products | 0.01 | 0.01, 0.02 |
| 247 | Other fruiting vegetables | 0.04 | 0.02, 0.04 |
| 248 | Other vegetables and vegetable combinations | 0.2 | 0.1, 0.2 |
| 249 | Dishes where vegetable is the major component | 0.2 | 0.1, 0.4 |
| 25 | **Legume and pulse products and dishes** | 0.04 | 0.01, 0.07 |
| 251 | Mature legumes and pulses | 0.004 | 0.00 ^a^, 0.007 |
| 252 | Mature legume and pulse products and dishes | 0.04 | 0.01, 0.07 |
| 26 | **Snack foods** | 0.3 | 0.2, 0.5 |
| 261 | Potato snacks | 0.05 | 0.03, 0.08 |
| 262 | Corn snacks | 0.05 | 0.03, 0.08 |
| 263 | Extruded or reformed snacks | 0.11 | 0.05, 0.18 |
| 264 | Other snacks | 0.15 | 0.05, 0.26 |
| 27 | **Sugar products and dishes** | 0.3 | 0.2, 0.4 |
| 271 | Sugar, honey and syrups | 0.02 | 0.01, 0.04 |
| 272 | Jam and lemon spreads, chocolate spreads, sauces | 0.05 | 0.03, 0.07 |
| 273 | Dishes and products other than confectionery where sugar is the major component | 0.2 | 0.1, 0.3 |
| 28 | **Confectionery and cereal/nut/fruit/seed bars** | 0.5 | 0.3, 0.7 |
| 281 | Chocolate and chocolate-based confectionery | 0.4 | 0.3, 0.6 |
| 282 | Fruit, nut and seed-bars | 0.004 | 0.001, 0.008 |
| 283 | Muesli or cereal style bars | 0.05 | 0.03, 0.06 |
| 284 | Other confectionery | 0.01 | 0.01, 0.01 |
| 29 | **Alcoholic beverages** | 0.00098 | 0.00 ^a^, 0.0023 |
| 291 | Beers | 0 |  |
| 292 | Wines | 0.00098 | 0.00 ^a^, 0.0023 |
| 293 | Spirits | 0 |  |
| 294 | Cider and perry | 0 |  |
| 295 | Other alcoholic beverages | 0 |  |
| 30 | **Special dietary foods** | 0.1 | 0.00 ^a^, 0.3 |
| 301 | Formula dietary foods | 0.1 | 0.00 ^a^, 0.3 |
| 31 | **Miscellaneous** | 0.10 | 0.07, 0.13 |
| 311 | Yeast, and yeast vegetable or meat extracts | 0.10 | 0.10, 0.13 |
| 312 | Intense sweetening agents | 0 |  |
| 313 | Herbs, spices, seasonings and stock cubes | 0.002 | 0.00 ^a^, 0.005 |
| 314 | Essences | 0.0001 | 0.00 ^a^, 0.0002 |
| 315 | Chemical raising agents and cooking ingredients | 0 |  |
| 32 | **Infant formulae and foods** | 0.8 | 0.2, 1.3 |
| 321 | Infant formulae and human breast milk | 0.7 | 0.2, 1.3 |
| 322 | Infant cereal products | 0.0001 | 0.00 ^a^, 0.0004 |
| 323 | Infant foods | 0.0026 | 0.00 ^a^, 0.0056 |
| 324 | Infant drinks | 0.003 | 0.00 ^a^, 0.009 |

^a^ Negative lower bounds were reported as 0.00, as negative dietary contributions are not plausible.

**References**

1. Food Standards Australia New Zealand (2016) AUSNUT 2011-13. Australian Government. <https://data.gov.au/data/dataset/ausnut-2011-13>. Accessed 5 May 2025.
